# How do face masks impact communication amongst deaf/HoH people?

**DOI:** 10.1186/s41235-022-00431-4

**Published:** 2022-09-05

**Authors:** Eva Gutierrez-Sigut, Veronica M. Lamarche, Katherine Rowley, Emilio Ferreiro Lago, María Jesús Pardo-Guijarro, Ixone Saenz, Berta Frigola, Santiago Frigola, Delfina Aliaga, Laura Goldberg

**Affiliations:** 1 Department of Psychology, University of Essex, Wivenhoe Park, Colchester, CO4 3SQ UK; 2 DCAL Research Centre, University College London, London, UK; 3 Universidad de Castilla-La Mancha, Cuenca, Spain; 4 University Pompeu Fabra, Barcelona, Spain; 5 Barcelona, Spain

**Keywords:** Deafness, COVID-19, Masks, Transparent masks, Communication, Wellbeing, Accessibility

## Abstract

Face coverings have been key in reducing the spread of COVID-19. At the same time, they have hindered interpersonal communication, particularly for those who rely on speechreading to aid communication. The available research indicated that deaf/hard of hearing (HoH) people experienced great difficulty communicating with people wearing masks and negative effects on wellbeing. Here we extended these findings by exploring which factors predict deaf/HoH people’s communication difficulties, loss of information, and wellbeing. We also explored the factors predicting perceived usefulness of transparent face coverings and alternative ways of communicating. We report the findings from an accessible survey study, released in two written and three signed languages. Responses from 395 deaf/HoH UK and Spanish residents were collected online at a time when masks were mandatory. We investigated whether onset and level of deafness, knowledge of sign language, speechreading fluency, and country of residence predicted communication difficulties, wellbeing, and degree to which transparent face coverings were considered useful. Overall, deaf/HoH people and their relatives used masks most of the time despite greater communication difficulties. Late-onset deaf people were the group that experienced more difficulties in communication, and also reported lower wellbeing. However, both early- and late-onset deaf people reported missing more information and feeling more disconnected from society than HoH people. Finally, signers valued transparent face shields more positively than non-signers. The latter suggests that, while seeing the lips is positive to everyone, signers appreciate seeing the whole facial expression. Importantly, our data also revealed the importance of visual communication other than speechreading to facilitate face-to-face interactions.

**Supplementary Information:**

The online version contains supplementary material available at 10.1186/s41235-022-00431-4.

## Highlights

- Late-onset deaf people experienced more difficulties in communication and low wellbeing.
- Severely/profoundly deaf people missed more information and felt disconnected from society.
- Signers preferred completely transparent face coverings.
- More frequent use of masks doesn’t necessarily imply more difficulty communicating.
- Visual communication, pro-social behaviour, and societal structure might help easing communication.

**Supplementary Information:**

The online version contains supplementary material available at 10.1186/s41235-022-00431-4.

## Significance statement

The present study is a tight collaboration between deaf and hearing researchers in the fields of cognitive psychology and language processing in deaf people. We investigate the communication challenges faced by deaf and hard of hearing (HoH) people due to the community-wide use of face masks to help stopping the spread of COVID-19. Previous knowledge of basic research on language processing in deaf people informed both the survey’s design and the selection of predictors for the regression analyses. For example, level and onset of deafness were included as separate predictors because basic research shows that they result in different levels of reliance on speechreading to aid communication. Similarly, sign languages (SLs) are the first language of many deaf/HoH people. Knowing a SL could equip the signer with visual-communication strategies other than speechreading.

Our findings highlight the importance of acknowledging the diversity in the deaf/HoH population and hence tailor interventions to their specific needs. We found that late-onset severely and profoundly deaf people experienced more difficulties communicating and lower wellbeing. However, it was signers who thought they had missed more information and felt more disconnected from society. Clear window masks, while better than opaque masks for all deaf/HoH people, might be most effective for fluent speechreaders and for non-signers. Interactions with signers could be improved by using completely transparent face coverings, paired with increased ventilation and social distancing for safety. Finally, other ways of communication such as using SL as much as possible, gesturing, writing or a combination of them can be particularly useful for deaf people.

## Introduction

Face masks have been shown to effectively reduce the risk of COVID-19 infection (Chu et al., 2020). This meant that by the summer of 2020 most countries required use of masks in public locations (Howard et al., 2021). Furthermore, it is likely that the use of face coverings will persist to some degree in the near future (Ballard, 2022; BBC, 2021; Wisconsin Public Radio, 2022). Facial coverings have been essential to control the spread of the virus and therefore to save lives. At the same time, masks hinder interpersonal communication as they attenuate the vocal sounds and cover facial expressions and lip movements (Mheidly et al., 2020). Masks add to the communication challenges faced by deaf and hard-of-hearing (HoH) people, who often rely on speechreading (lipreading^1^) to aid communication. The deaf and HoH communities warned early in the pandemic that use of face masks was likely to have a negative impact on communication (see e.g. jewishnews.timesofisrael.com, 2020; Lavanguardia.com, 2021; NDCS, 2021; RNID, 2021b; Saiz et al., 2022) because it impedes speechreading. Similar warnings were issued from the medical community (Chodosh et al., 2020), highlighting that masks negatively affect speech perception in hospitals (particularly for older people) and that the lack of visual cues from the face was likely to have a major impact on deaf people (see Tavanai et al., 2021). The present survey study investigated the impact of face masks on loss of information, wellbeing, and how communication could be improved.

According to the World Health Organisation (WHO), approximately 430 million people worldwide have hearing loss (WHO, 2021), 12 million in the UK (RNID, 2021a), and over one million in Spain (see e.g. INE, 2021). It is well known that the deaf and HoH population is extremely heterogeneous. For example, in the UK approximately 28% of deaf/HoH people became deaf/HoH at birth or early childhood, and around 71% became deaf/HoH later in life, most of them as a result of ageing (see e.g. ONS, 2018). People with different onset of deafness are likely to adopt different communication strategies (Garstecki & Erler, 1999). For instance, it has been shown that people with late-onset deafness might be reluctant to disclose their deafness, reducing the number of social interactions they experience altogether (Barker et al., 2017). Conversely, early-onset deaf people are likely to have developed more efficient visual communication strategies (including learning sign language, developing better speechreading ability, use of manually coded speech, cued speech, increased use of pointing and gesturing, etc., see e.g. Gravel & O'Gara, 2003; Mohammed et al., 2006). A second source of differences is linked to the amount of speech signal that can be accessed depending on the level of deafness. Even individuals with mild or moderate hearing loss (referred through this paper as HoH) often miss most consonants and experience difficulty perceiving vowel sounds (see e.g. Northern & Downs, 2002). On the other hand, speech can be largely inaudible without hearing aids to people with severe or profound hearing loss (referred through this manuscript as deaf; see e.g. Northern & Downs, 2002; Olusanya et al., 2019). There are also differences in age of first language acquisition and language fluency in either spoken or sign language. For example, it has been found that children with mild to severe deafness, who often are not exposed early to a sign language, show delays in spoken language development (Tomblin et al., 2015). However, deaf children who have been exposed to an accessible visual language early in life are much less likely to show the detrimental effects of language deprivation (see e.g. Humphries et al., 2012; Kushalnagar et al., 2010).

Another critical source of differences is the amount of information that can be accessed through speechreading in interactions without masks, with only a proportion of deaf/HoH people being fluent speechreaders. With only 30–40% of speech sounds distinguishable on the lip-patterns (Woodward & Barber, 1960), proficient access to speech through speechreading is challenging for many deaf/HoH people. All these differences lead to highly variable everyday communication experiences that can be affected differently by the use of face masks. Finally, another factor relevant to everyday communication while using face coverings could be the cross-cultural differences affecting the frequency with which masks have been used and the development of communication alternatives. Here we explore the impact of mask usage on deaf/HoH people in two countries where regulations about mandatory mask usage was distinct: Spain and the UK. In Spain, using face masks was mandatory at the time of the survey (November 2020 to February 2021) in public in both indoor and outdoor spaces and with very few exemptions allowed (e.g. with an updated certificate from a medical practitioner justifying the exemption). In the UK, outdoor mask use was not mandatory, and there were more exemptions compared to Spain. These differences, together with other possible cultural differences (i.e. more generalised use of gestures accompanying speech in Spain), could have led to different communicative experiences. In summary, in addition to country of residence, we study whether onset and level of deafness, knowledge of sign language, and speechreading ability predict respondents’ perceptions of difficulty in communication, impact on wellbeing, and efficacy of clear view face coverings.

## Communication difficulties

A handful of survey studies run by organisations working with deaf/HoH people reported that 85% of deaf/HoH respondents saw face coverings as an impediment for speechreading, and 72% of HoH respondents thought that masks made it more difficult for them to use their residual hearing to aid speech comprehension (Ideas for Ears, 2020). Market research from the hearing aids industry found that masks made communication more difficult and contributed to an increase in frustration, embarrassment, and isolation amongst the hearing aid users that answered their survey (Specsavers, 2021). Trecca et al. (2020) reported preliminary data from 59 Italian hospital patients with mild to profound hearing loss. The patients included in this study expressed concerns about the sound attenuation (44% of respondents) and the impossibility of speechreading when medical practitioners were using masks (56% of respondents). A survey conducted before masks were mandatory in the UK found that deaf/HoH people were concerned that the use of face coverings was becoming more common, with a trend for people with more severe levels of deafness to express higher levels of concern (Naylor et al., 2020). Another study conducted in the UK (Saunders et al., 2021) found that face coverings had a more negative impact in communication for deaf/HoH than for hearing people. In an online survey study of over 400 deaf/HoH people that was analysed using a combination of descriptive statistics and thematic analyses, Saunders et al. (2021) found detrimental effects of face coverings in the quality of hearing and understanding, significantly more for people with poor/very poor self-reported hearing. Furthermore, communication with masks was perceived by most participants as fatiguing and frustrating. The current study aims to build up on these previous results by exploring how the above predictors influence—if at all—the perceived difficulty of communicating. Saunders et al. (2021) also reported that the impact of face coverings might depend on the conversational situation, having a larger negative impact on medical settings or at work than in communication with family and friends. Here, besides a general measure of difficulty communicating when the conversational partner was using a mask, we added specific questions about professional as well as social situations.

## Loss of information and wellbeing

The increase in the use of face masks is likely to result in loss of information conveyed in interactions using masks. Studies carried out in medical settings pre-COVID, where masks were used but not as extensively, indicate that deaf/HoH people reported having missed considerable amounts of relevant information. In a study with 95 deaf/HoH university students in the UK and Ireland, Henn et al. (2021) found that 60% of participants experienced miscommunication during consultations that involved diagnosis, advice, and information about medication. Importantly, speaking while wearing surgical masks was one of the factors linked to miscommunications during consultations. Here we study whether or not the amount of information that deaf/HoH people thought they missed is linked to the above predictors.

While being hard for everyone (Epifanio et al., 2021; Ping et al., 2020), the community-wide use of face masks could have important consequences on deaf/HoH people’s wellbeing. Even before the COVID outbreak, research showed that deaf people experience numerous communication challenges, which have been linked to poorer mental health and lower quality of life (e.g. Margaret du Feu, 2014). One important reason why communication issues specific to face masks could have disproportionate consequences for deaf/HoH people is that many of them might rely predominantly on speechreading. Previous research has shown that deaf/HoH people are, as a group, more proficient speechreaders than hearing people (Mohammed et al., 2006), possibly having developed over time more efficient strategies to utilise visual speech information. For example, Kyle et al. (2013) found no differences in speechreading ability between hearing and early-onset deaf children. However, Mohammed et al. (2006) found better speechreading skill in a group of adult early-onset deaf people when compared to hearing people. These findings suggest a developmental trajectory in which deaf/HoH people improve their speechreading skill as a consequence of communication experience. Another reason why face covering might affect deaf/HoH people more is that mouth patterns and facial expressions are essential for sign language comprehension (see e.g. Sutton-Spence & Woll, 1999). Therefore, signers are likely to also be affected by the use of masks. Despite the importance of studying the effects of face masks on deaf/HOH people’s wellbeing, the number of empirical studies is extremely low. Saunders et al. (2021) found that for the deaf/HoH people in their study, the communication issues produced a number of negative emotions that included increased anxiety and feelings of isolation, as well as loss of confidence. Face coverings were also related with less willingness to engage in conversations and lower feeling of personal connection with the conversational partner. Here we explore whether the above predictors are linked to emotional wellbeing (measured as feeling of disconnection from society) and a general measure of quality of life that includes both mental and physical health. Our findings could reveal areas of vulnerability and thus contribute to more finely tuned guidelines offered to the deaf/HoH communities.

## Ways to improve communication

It is important to note that communication success largely depends on the conversational partner. Whether or not the interlocutor is deaf/HoH aware, how much gesture/body language they use, if they know sign language or not, whether or not they have an accent that the deaf/HoH person is familiar with, and how clear their mouth patterns are (e.g. Middleton et al., 2010; Smiljanić & Bradlow, 2009). Middleton et al. (2010) found that for communication success in clinical settings it is crucial that healthcare providers are deaf aware, and fluent signers are present if contact with deaf/HoH patients is common. Saunders et al. (2021) study revealed that deaf/HoH people adapted their own behaviour to include more gestures and explicit facial expressions (particularly using their eyes) to improve communication. In the present study we extended this question to the behaviours of the communication partners by asking deaf/HoH respondents to what degree they had observed other people’s attempts to communicate in an alternative way.

To facilitate speechreading, the healthcare community has advocated for the use of transparent face masks (see e.g. Chodosh et al., 2020). Likewise, some members of the deaf/HoH communities have petitioned for a wider use of communication friendly face coverings such as masks with a clear window (for examples of deaf people’s requests for clear face windows see e.g. Ideas for ears, 2020; NDCS, 2020). However, other deaf/HoH people have warned that transparent face masks might not resolve communication issues entirely (for an example in Spanish see Emilio Ferreiro, 2020), arguing that there might be large differences between deaf/HoH people in the amount of information they can extract from lip patterns. Published research seems to support this view. Take for example Mohammed et al’s. (2006) study where deaf participants were not only significantly better speechreaders than hearing participants at the group level, but a large variability in speechreading skill can also be observed in the study’s participants (Mohammed et al., 2006; Fig. 3). Furthermore, transparent face coverings are expensive, there are few manufacturers available, and manufacturing is not yet regulated (Chodosh et al., 2020). Research investigating to what extent transparent face coverings facilitate communication, and what groups are more likely to benefit from seeing the lips is still scarce. A pre-COVID study (Atcherson et al., 2017) found that deaf/HoH people who were trying to understand speech in noise performed best in the transparent than in the standard mask condition. Recently, Homans and Vroegop (2022) also found that transparent face shields, despite distorting the acoustic signal more than surgical masks, led to better speech understanding. Similarly, Saunders et al. (2021) also found that deaf/HoH respondents evaluated transparent face covering positively. Here we investigate deaf/HoH people’s perceptions of both clear window masks and transparent face shields.

## Current research

The empirical studies available seem to converge on the increased communication challenges imposed by masks for deaf/HoH people in general, both in terms of increased difficulty and detrimental effects on wellbeing. However, research is needed into what aspects of deafness, if any, play a bigger role during communication using face coverings. Additionally, little is known about how different regulations regarding mandatory use of masks have had an impact on communication. Finally, it has been suggested that transparent face coverings could have a beneficial effect for deaf/HoH people but there remains no research into who might benefit more from communication friendly face masks. Here we report an exploratory survey study that investigates which factors predict communication difficulties, wellbeing, and usefulness of transparent face coverings. Importantly, the present study has a strong focus on accessibility. In order to conduct research that represents well the deaf/HoH populations, we provided participants with the opportunity to see the survey in their preferred language. Therefore, we released our survey not only in two written languages but also 3 different sign languages (British sign language [BSL], Spanish sign language [lengua de signos española: LSE] and Catalan sign language [llengua de signes catalana: LSC]. Finally, the data for the present study was collected between November 2020 and February 2021, when face masks had been mandatory and widely used both in Spain and the UK for several months (Table 1).

**Table 1:** Summary of the current research

Survey languages	Predictors	Outcome measures
Written English	Level of deafness	General difficulty communicating with others who wear masks
Written Spanish	Onset of deafness	Difficulty communicating with others who wear masks in professional situations
British Sign Language (BSL)	Knowledge of sign language	Difficulty communicating with others who wear masks in informal social situations
Spanish Sign Language (Lengua de signos Española: LSE)	Lipreading fluency	Perceived amount of information missed
Catalan Sign Language (llengua de signes catalana: LSC)	Country of residence	Feeling of disconnection from society
		General wellbeing (quality of life)
		Perceived efforts made by others to improve communication
		Perceived efficacy of clear masks
		Perceived efficacy of transparent face shields

## Methods

### Participants

Only complete datasets from deaf or HoH people living in Spain and the UK were downloaded from the Qualtrics platform and analysed (an additional 109 datasets corresponded to clicks on the survey link whereafter clicking consent and choosing language no other question was answered). A total of 395 deaf or HoH people from the UK (n = 273) and Spain (n = 122) voluntarily completed this survey in exchange for participation in a prize draw. Participants were on average 45.6 years old (*SD* = 15.9, range 18–81), respondents’ characteristics are shown in Table 2. One hundred and twelve deaf respondents (28.4% of the total sample) completed the sign language version of the survey. After they had given their answers, participants were offered the option to enter one of 6 prize draws (£50 or 50€ depending on their country of residency). Participants were recruited through word of mouth, social media posts, and deaf organisations email distribution lists. This study was approved by University of Essex Science and Health Ethics Sub-committee (ETH2021-0196).

**Table 2:** Participants’ characteristics

	UK		Spain		Total	
	*n*	%	*n*	%	*n*	%
*Gender*						
Female	195	**49.4**	78	**19.7**	273	**69.1**
Male	74	**18.7**	39	**9.9**	113	**28.6**
Genderqueer			2	**0.5**	2	**0.5**
Transgender	1	**0.3**			1	**0.3**
Prefer not to say	3	**0.8**	3	**0.8**	6	**1.5**
Cochlear implant fitted	52	**13.2**	33	**8.4**	85	**21.5**
Belongs to a vulnerable group	89	**22.5**	28	**7.1**	117	**29.6**
Had COVID-19	29	**7.3**	13	**3.3**	42	**10.6**
*Level of deafness*						
Hard of hearing	79	**20.0**	28	**7.1**	107	**27.0**
Deaf	194	**49.1**	94	**23.8**	288	**73.0**
*Onset of deafness*						
Early	179	**45.3**	94	**23.8**	273	**69.1**
Late	96	**24.3**	26	**6.6**	122	**30.9**
*Knowledge of SL*						
Signer	198	**50.1**	87	**22.0**	285	**72.2**
Non-signer	74	**18.7**	35	**8.9**	109	**27.8**
*Lipreading fluency*						
Fluent	183	**46.3**	91	**23.0**	274	**69.4**
Non-fluent	85	**21.5**	27	**6.8**	112	**28.4**
Total	273	**68.9**	122	**30.8**	395	**100**

Percentages are bolded

### Procedure

After giving informed consent, participants answered the survey items online, using their own devices and at their own pace. Participants were allowed 1 week to complete the survey; previous responses were saved as long as they accessed the link using the same device. Data was collected between the 3rd of November 2020 and the 10th of February 2021, while the use of face masks was mandatory in both countries (although with different regulations regarding indoors/outdoors wearing).

### Materials

Our survey included demographic questions (e.g. age, gender, country of residence, etc.) as well as the following deafness and language background measures (predictors in the regression analyses, together with country of residence):

#### Country of residence

For the analyses, country of residence was vector coded − 1 = Spain and 1 = UK.

#### Level of deafness

Participants answered to the question “What is the level of deafness in your best ear?” they selected one of five options (mild, moderate, severe, profound, I don’t know). We considered those who reported either mild or moderately deaf in their best ear to be HoH. We considered those who reported either severely or profoundly deaf in their best ear to be deaf, except for 5 who did not know. These 5 participants who answered “I don’t know” to this item were categorised as deaf based on their answers to other background questions (e.g. they currently are or had been users of a cochlear implant, which is only offered to people with severe to profound deafness). This distinction is important because HoH people have some access to speech sounds without aids, while severely or profoundly deaf people generally have very little or no access to speech sounds without aids. For the analyses, level of deafness was vector coded − 1 = HoH and 1 = deaf.

#### Onset of deafness

People that became deaf at age 10 or older were considered late-onset, while people becoming deaf before the age of 9 years old were considered early-onset. This distinction was based on previous studies with deaf people who considered age 9 as the cut-off age for early language development (for a recent review see Mayberry & Kluender, 2018). In our sample, 228 out of 276 early-onset respondents were deaf at birth or before 3 years of age, only 9 reported becoming deaf between the ages of 6 and 9. Finally, 108 out of the 120 late-onset respondents became deaf after the age of 15. For the analyses, onset of deafness was vector coded − 1 = Late and 1 = early.

#### Knowledge of SL

Participants answered yes if they knew any sign language and no if they did not know any sign language. For the analyses, Knowledge of SL was coded − 1 = non-signer and 1 = signer.

#### Lipreading fluency

Participants were considered non-fluent if they responded average, poor, or not at all at the question “Indicate how fluent you are—that is how well you are able to communicate—in Spoken English: lipreading” (in the Spanish version: “Indicate how fluent you are—that is how well you are able to communicate—in Spoken Spanish: lipreading”). If their answer was I don’t know it at all, poor, or average they were considered non-fluent. Conversely, if their answer was good, excellent, or native-like they were considered fluent at lipreading. For the analyses, lipreading fluency was vector coded − 1 = non-fluent and 1 = fluent.

#### Measures of mask usage

We also included questions aimed to explore the frequency of their experiences in communication settings using face masks. Specifically, respondents answered, on a scale of 1 (never) to 5 (always), how often masks were worn outside of their own house by themselves, their family members, their deaf friends, and their hearing friends. They also answered how many hours a day they communicated with other people that were wearing a mask. The response options (1–4) were *“less than 1 h a day”*, *“1–3 h a day”, “4–6 h a day”* and *“more than 6 h a day”*.

Finally, the dependent measures of interest were the following:

##### General difficulty communicating with others who wear a mask

Participants answered the question *“In general, how easy do you think communication is when other people wear a face mask?*” the response options (1–5) were, “1 = *very difficult”, “2* = *difficult”, “3* = *neither difficult or easy”, “4* = *easy”*, “5 = *very easy”*. Responses were reverse coded for analyses.

##### Difficulty communicating with others who wear a mask in professional situations

This measure reflects the average of the inverse coded responses to the question *“How easy do you think communication is when other people wear a face mask in the following situations?”* in the following items: *Hospital or doctor’s appointments, Education/courses, Workplace, Shopping, official interactions (banks, bills, council, *etc*.;* Cronbach’s alpha = 0.808). The response options (1–5) were, “1 = *very difficult”, “2* = *difficult”, “3* = *neither difficult or easy”, “4* = *easy”*, “5 = *very easy”*.

##### Difficulty communicating with others who wear a mask in informal social situations

This measure reflects the average of the inverse coded responses to the question *“How easy do you think communication is when other people wear a face mask in the following situations?”* in the following items: Communication with friends, communication with romantic partner, and communication with family (Cronbach’s alpha = 0.913). The response options (1–5) were, “1 = *very difficult”, “2* = *difficult”, “3* = *neither difficult or easy”, “4* = *easy”*, “5 = *very easy”*.

##### Amount of information missed

Participants answered the question *“Since the COVID-19 outbreak, how much information do you think you have missed when communicating with people while they were wearing a mask and speaking? “* the response options (1–5) were* “1* = *I have missed no information at all”, “2* = *I have missed a little information”, “3* = *I have missed a moderate amount of information”, “4* = *I have missed a lot of information”,* and *“5* = *I have missed a great deal of information”*.

##### Emotional wellbeing: feeling of disconnection from society

Participants answered the question *“Since the COVID-19 outbreak how much of the time have you felt more disconnected from society than before?”* the response options (1–6) were “1 = *none of the time”, “2* = *a little of the time”, “3* = *some of the time”, “4* = *a good bit of the time”*, *“5* = *most of the time”,* and “6 = *all of the time”*.

##### General wellbeing: quality of life

We used the SF-12 questionnaire v.1 (Ware Jr et al., 1996) to measure quality of life in the 4 weeks prior to answering the survey. The SF-12 assess the impact of health on someone’s everyday life that is often used as a wellbeing measure. The original SF-12 questionnaire has been translated to multiple languages, including Spanish (e.g. Vilagut et al., 2008) but not to any sign language. For the purposes of this research, we translated to BSL, LSE and LSC the questionnaire items (see details below). Since this questionnaire has not been validated with deaf and HoH populations, for the purposes of this study we use the raw scores in the analysis.

##### Perception of others’ efforts to improve communication

Participants answered the question *“In general, if you were missing some information when communicating with people that wore a mask and spoke, how much did they engage in alternative ways of communication (e.g. gesturing, writing, using the mobile phone, *etc*.)?”* the response options (1–5) were *“1* = *Not at all”, “2* = *A little”, “3* = *A moderate amount”, “4* = *A lot”,* and *“5* = *A great deal”*.

##### Perceived efficacy of clear window masks

Participants answered the question *“How much do you think that face masks with a clear window help to solve communication issues?”* We used the same response options included in the others’ efforts to communicate measure.

##### Perceived efficacy of transparent face shields

Participants answered the question *““How much do you think that transparent face shields help to solve communication issues?”* We used the same response options included in the others’ efforts to communicate measure.

All survey items were developed in English and translated to written Spanish, British Sign Language (BSL), Spanish sign language (LSE) and Catalan Sign Language (LSC). We followed a back translation procedure in which, one proficient user of 2 languages translated all items from English to written Spanish and to BSL and from written Spanish to LSE and LSC. Several different proficient users of two of the languages, who had not seen the original items, then back-translated the written Spanish and BSL questions to English and the LSE and LSC questions to written Spanish. This procedure was repeated, by different independent translators with no knowledge of the previous versions, until congruency was achieved between the original and back translated items. There were 10 people involved in translations in total, 3 from English to Spanish, 2 from Spanish to LSE, 2 from English to BSL and 3 from Spanish to LSC.

Deaf native signers of BSL, LSE and LSC were filmed producing each survey item. We used a HD recording device, a greenscreen background, and professional illumination. To ensure consistency, we kept filming conditions, including model’s clothes and physical appearance the same across the different recording sessions. Videos were edited offline so they all had a neutral background, the signer could be seen clearly, and each video clip contained the survey question and the possible options. The signed version was always presented together with the written text. Participants answered by clicking on the written response but note that the exact position of each response option had been described in the video, right after the question, and that the video could be repeated on demand. The response options were presented with the question to avoid technical difficulties and/or extra effort due to clicking on one independent video for each answer type.

## Results

**Fig. 1:**
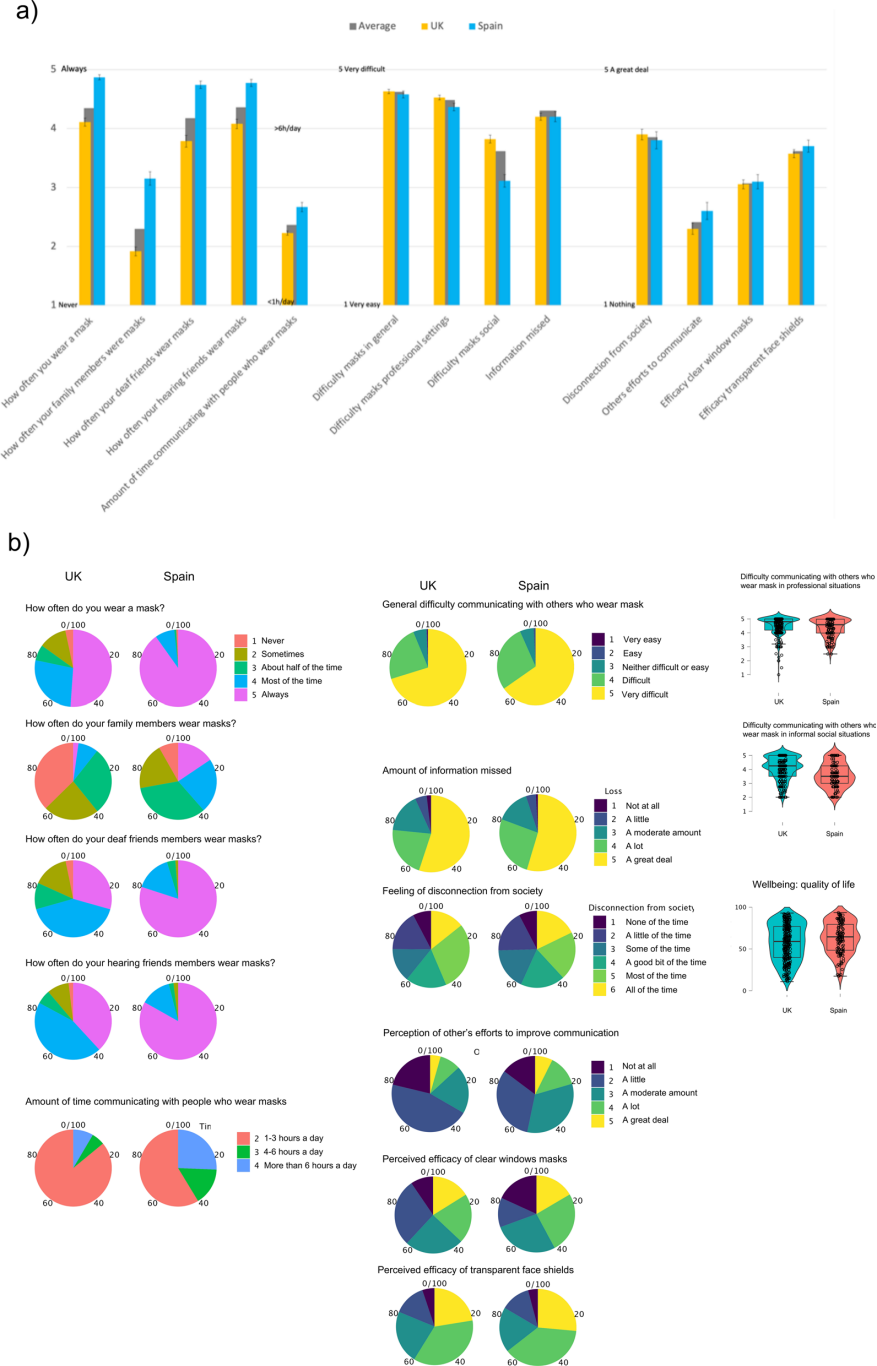
**a** Mean responses to measures assessing frequency of interactions involving masks and to the study’s dependent variables. Mean values are shown for UK and Spanish residents separately, the grey bar in the background corresponds to the total average. **b** The distribution of responses to the same measures, a violin plot rather than a pie chart has been used for those measures that comprise continuous values

In order to ensure that respondents had experience in communication settings using face masks, we first carried out one-sample *t*-tests on the measures of frequency of mask wearing (see Table 3; see also Fig. 1 for mean and distribution of responses). Results revealed that on average the participants and their deaf and hearing friends wore masks *most of the time*. Their family members wore masks on average *some of the time*. Finally, on average participants spent around 1 and 3 h a day communicating with people who wear masks. The values for all measures were significantly higher than the lowest possible value, indicating that respondents had participated often in communication settings where face masks were worn. This pattern of results was the same when residents in the UK and Spain were considered separately (all *p*s < 0.001, all Cohen’s *d* > 2.2). However, independent samples *t*-tests comparing UK and Spanish residents showed that Spanish residents wore masks more often than UK residents, *t* (392) = 6.97 *p* < 0.001. Spanish residents’ interlocutors also wore masks more often than UK residents’ interlocutors: family members, *t*(393) = 8.96 *p* < 0.001, deaf friends, *t* (207) = 7.23 *p* < 0.001, and hearing friends, *t*(251) = 6.33 *p* < 0.001. Finally, Spanish residents spent more time communicating with people wearing masks than UK residents, *t*(390) = 5.94 *p* < 0.001.

Then we carried out similar one-sample *t*-tests on the study-dependent variables (except for the wellbeing measure for which we did not have an appropriate standardised value to test against since it has not been validated in this population before). The results shown in Table 3 and in Fig. 1 indicate that on average, respondents thought that communication was “*very difficult*” in general and in professional settings, and “*difficult*” in informal social settings. They thought they had missed “*a lot of information*” in these interactions; they have felt disconnected from society “*a good bit of the time*”. On average, respondents thought that when they were experiencing difficulties in communication, people wearing masks engaged in alternative ways of communication “*a little*”. Finally, respondents thought that clear window masks help to solve communication problems “*a moderate amount*”, while transparent face shields help “*a lot*”. The values for all measures were significantly higher than the lowest possible value, indicating that respondents had significantly experienced communication difficulties, lack of information, and disconnection from society and at the same time, some efforts from others to communicate in alternative ways as well as the benefits of seeing their interlocutor’s lip patterns. This pattern of results was the same when residents in the UK and Spain were considered separately (all *p*s < 0.001, all Cohen’s *d* > 1.5).

**Table 3:** Average values and one sample *t*-tests for frequency of mask use and dependent variables

	Mean	SD	*t*	*df*	*p*	Cohen’s *d*
How often you wear a mask (1-never → 5-always)	4.3	1.1	62.8	394	< .001	4.11
How often your family members were masks (1-never → 5-always)	2.3	1.4	18.5	395	< .001	1.65
How often your deaf friends wear masks (1-never → 5-always)	4.2	1.1	43.8	210	< .001	3.97
How often your hearing friends wear masks (1-never → 5-always)	4.4	0.9	58.2	252	< .001	4.75
Amount of time communicating with people who wear masks (1- < 1 h a day → 4 - > 6 h a day)	2.4	0.7	37.9	392	< .001	3.32
Difficulty masks in general (1-very easy → 5-very difficult)	4.6	0.6	112.0	393	< .001	7.20
Difficulty masks professional settings (1-very easy → 5-very difficult)	4.5	0.7	100.2	392	< .001	6.51
Difficulty masks social (1-very easy → 5-very difficult)	3.6	1.2	44.2	380	< .001	3.13
Information missed (1-nothing → 5-a great deal)	4.3	1.0	64.6	384	< .001	4.31
Disconnection from society (1-not at all → 5-all of the time)	3.8	1.6	36.3	391	< .001	2.48
Others’ efforts to communicate (1-not at all → 5-a great deal)	2.4	1.1	26.1	395	< .001	2.24
Efficacy clear window masks (1-not at all → 5-a great deal)	3.1	1.3	32.5	394	< .001	2.43
Efficacy transparent face shields (1-not at all → 5-a great deal)	3.6	1.1	45.9	393	< .001	3.20

Student’s *t*-test, the alternative hypothesis specifies that the mean is greater than 1

**Fig. 2:**
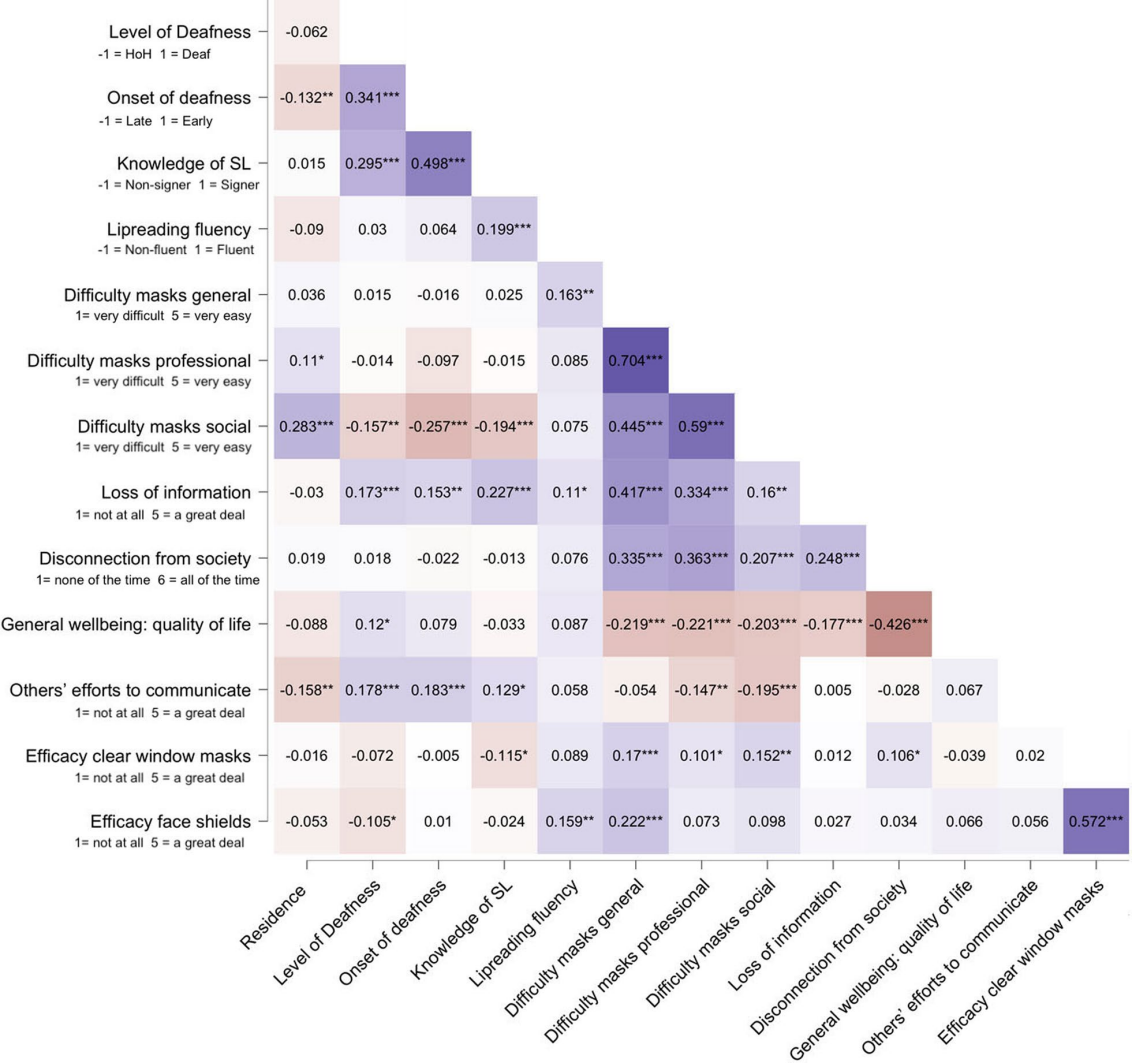
Heatmap showing the correlation between the different measures. Darker red colours represent a larger negative correlation while darker purple show a larger positive correlation

To study to what extent country of residence (− 1 = Spain, 1 = UK), level of deafness − 1 = HoH and 1 = deaf), onset of deafness (− 1 = late, 1 = early), knowledge of sign language (− 1 = non-signer, 1 = signer), and lipreading fluency (− 1 = non-fluent, 1 = fluent) predicted (A) experienced difficulty communicating with people wearing masks, (B) perceived loss of information and wellbeing and (C) opinion on ways to improve communication, we conducted a series of hierarchical regression analyses with the mentioned predictors and each of the dependent measures of interest. In the hierarchical regression the first step contained the main effects, successive steps (2 to 5) included all possible 2-way, 3-way, 4-way and 5-way interactions between predictors. There were no 4-way nor 5-way interactions (*p*s > 0.067), therefore we limit reporting to the highest-order significant interactions in Tables 4, 5, and 6 (the remaining statistics are shown in the Additional file 1). Significant interactions were decomposed to test for the simple effects using Aiken et al., (1991). For clarity, only the significant simple effects are reported in the text (the remaining statistics are shown in the Additional file 1). Figure 2 shows the correlations between the different predictors and outcome measures. Weak to moderate significant correlations can be observed between our predictors, indicating some degree of shared variance. For example, respondents with early-onset deafness also tended to be signers as well as deaf (as opposed of HoH). Deaf respondents also tended to be signers (as opposed to not knowing SL). Those who reported knowing sign language (signers) also reported being fluent lipreaders. Significant correlations can also be observed between the three different measures of difficulty communicating with others who wear masks and the rest of the outcome measures.

**Table 4:** Summary of significant effects in the regression analysis for experienced difficulty communicating with people who wear masks

Model		*b*	*t*	*p*	95% CI	
					*LL*	*UL*
*Outcome measure: general difficulty communicating with people wearing masks*						
1	Lipreading fluency	0.164	3.149	0.002	0.043	0.186
2	Residence * Lipreading fluency	0.171	2.679	0.008	0.029	0.188
2	Level deaf. * Onset deaf	− 0.167	− 2.795	0.005	− 0.202	− 0.035
*Outcome measure: difficulty communicating with people who wear masks in professional settings*						
1	Residence	0.104	2.014	0.045	0.002	0.155
2	Level deaf. * Onset deaf	− 0.125	− 2.072	0.039	− 0.190	− 0.005
3	Lipreading fluency	− 0.256	− 2.097	0.037	− 0.378	− 0.012
3	Residence * Lipreading fluency	0.219	2.312	0.021	0.023	0.282
3	Level deaf. * SL	− 0.194	− 1.996	0.047	− 0.298	− 0.002
*Outcome measure: difficulty communicating with people wearing masks in informal social situations*						
1	Onset deaf	− 0.144	− 2.500	0.013	− 0.321	− 0.038
1	SL	− 0.126	− 2.168	0.031	− 0.308	− 0.015
1	Lipreading fluency	0.140	2.831	0.005	0.054	0.302
2	Residence	0.199	3.178	0.002	0.095	0.404
2	Residence * Level deaf	− 0.142	− 2.130	0.034	− 0.318	− 0.013
2	Residence * SL	0.160	2.201	0.028	0.020	0.354
2	Level deaf. * Onset deaf	− 0.211	− 3.806	0.000	− 0.415	− 0.132
3	Onset deaf	− 0.271	− 2.076	0.039	− 0.660	− 0.018
3	Level deaf. * Onset deaf	− 0.185	− 1.996	0.047	− 0.476	− 0.003

CI = confidence interval; *LL* = lower limit; *UL* = upper limit. The predictors were vector coded: Residence: UK = 1, Spain =  − 1; Level deaf.: Deaf = 1, HoH =  − 1; Onset deaf.: Early = 1, Late =  − 1; SL: Know SL = 1, Does not know SL =  − 1; Lipreading fluency: Fluent = 1, non-fluent =  − 1

**Table 5:** Summary of significant effects in the regression analysis amount of missed information, feeling of being disconnected from society, and wellbeing

Model		*b*	*t*	*p*	95% CI	
					*LL*	*UL*
*Outcome measure: amount of information missed*						
1	Level deaf	0.120	2.230	0.026	0.016	0.250
1	SL	0.167	2.792	0.006	0.054	0.313
2	SL	0.219	2.439	0.015	0.047	0.435
2	Level deaf. * Onset deaf	− 0.146	− 2.443	0.015	− 0.291	− 0.031
*Outcome measure: feeling of being disconnected from society*						
2	Level deaf	0.180	2.292	0.022	0.044	0.579
2	Residence * Level deaf	− 0.218	− 2.952	0.003	− 0.565	− 0.113
2	Residence * SL	0.174	2.159	0.032	0.024	0.521
3	Level deaf	0.333	2.620	0.009	0.144	1.009
3	Residence * Level deaf	− 0.376	− 2.710	0.007	− 1.012	− 0.161
3	Residence * SL	0.286	2.113	0.035	0.031	0.865
3	Level deaf. * Onset deaf. * SL	− 0.151	− 2.003	0.046	− 0.502	− 0.005
*Outcome measure: quality of life*						
1	Level deaf	0.129	2.377	0.018	0.527	5.571
1	SL	− 0.157	− 2.580	0.010	− 6.514	− 0.879
1	Lipreading fluency	0.108	2.083	0.038	0.140	4.871
2	Onset deaf	0.223	2.429	0.016	0.973	9.248
2	SL	− 0.212	− 2.311	0.021	− 9.264	− 0.746
2	Lipreading fluency	0.174	2.449	0.015	0.796	7.275
2	Residence * Onset deaf	− 0.172	− 2.059	0.040	− 7.107	− 0.163
3	Onset deaf	0.325	2.246	0.025	0.927	13.962
3	Lipreading fluency	0.289	2.344	0.020	1.078	12.330
3	Residence * Level deaf	0.307	2.214	0.027	0.732	12.362
3	Level deaf. * Onset deaf	0.251	2.445	0.015	1.165	10.736

CI = confidence interval; *LL* = lower limit; *UL* = upper limit. The predictors were vector coded: Residence: UK = 1, Spain =  − 1; Level deaf: Deaf = 1, HoH =  − 1; Onset deaf.: Early = 1, Late =  − 1; SL: Know SL = 1, Does not know SL =  − 1; Lipreading fluency: Fluent = 1, non-fluent =  − 1

**Table 6:** Summary of significant effects in the regression analysis on measures related with communication improvement

Model		*b*	*t*	*p*	95% CI	
					*LL*	*UL*
*Outcome measure: perceived effort from others to improve communication*						
1	Residence	− 0.132	− 2.619	0.009	− 0.271	− 0.039
1	Level deaf	0.130	2.427	0.016	0.030	0.283
3	Residence * Onset deaf	− 0.318	− 2.237	0.026	− 0.644	− 0.041
*Outcome measure: perceived efficacy of transparent or clear window masks to facilitate communication*						
1	SL	− 0.179	− 2.950	0.003	− 0.419	− 0.084
1	Lipreading fluency	0.122	2.341	0.020	0.027	0.310
2	Onset deaf	0.180	1.983	0.048	0.002	0.491
2	SL	− 0.222	− 2.447	0.015	− 0.564	− 0.061
2	Residence * SL	0.238	3.075	0.002	0.110	0.499
2	Level deaf. * SL	− 0.166	− 2.491	0.013	− 0.417	− 0.049
3	SL	− 0.277	− 2.087	0.038	− 0.757	− 0.023
3	Residence * Level deaf	− 0.402	− 2.954	0.003	− 0.853	− 0.171
3	Residence * SL	0.389	2.935	0.004	0.164	0.832
3	Level deaf. * SL	− 0.216	− 2.248	0.025	− 0.569	− 0.038
3	Residence * Level deaf. * Lipreading fluency	0.295	2.268	0.024	0.050	0.696
*Outcome measure: perceived efficacy of transparent face shields to facilitate communication*						
1	Level deaf	− 0.107	− 1.974	0.049	− 0.269	− 0.001
1	Lipreading fluency	0.170	3.289	0.001	0.085	0.337

CI = confidence interval; *LL* = lower limit; *UL* = upper limit. The predictors were vector coded: Residence: UK = 1, Spain =  − 1; Level deaf: Deaf = 1, HoH =  − 1; Onset deaf.: Early = 1, Late =  − 1; SL: Know SL = 1, Does not know SL =  − 1; Lipreading fluency: Fluent = 1, non-fluent =  − 1

### A. Experienced difficulty communicating.

**Fig. 3:**
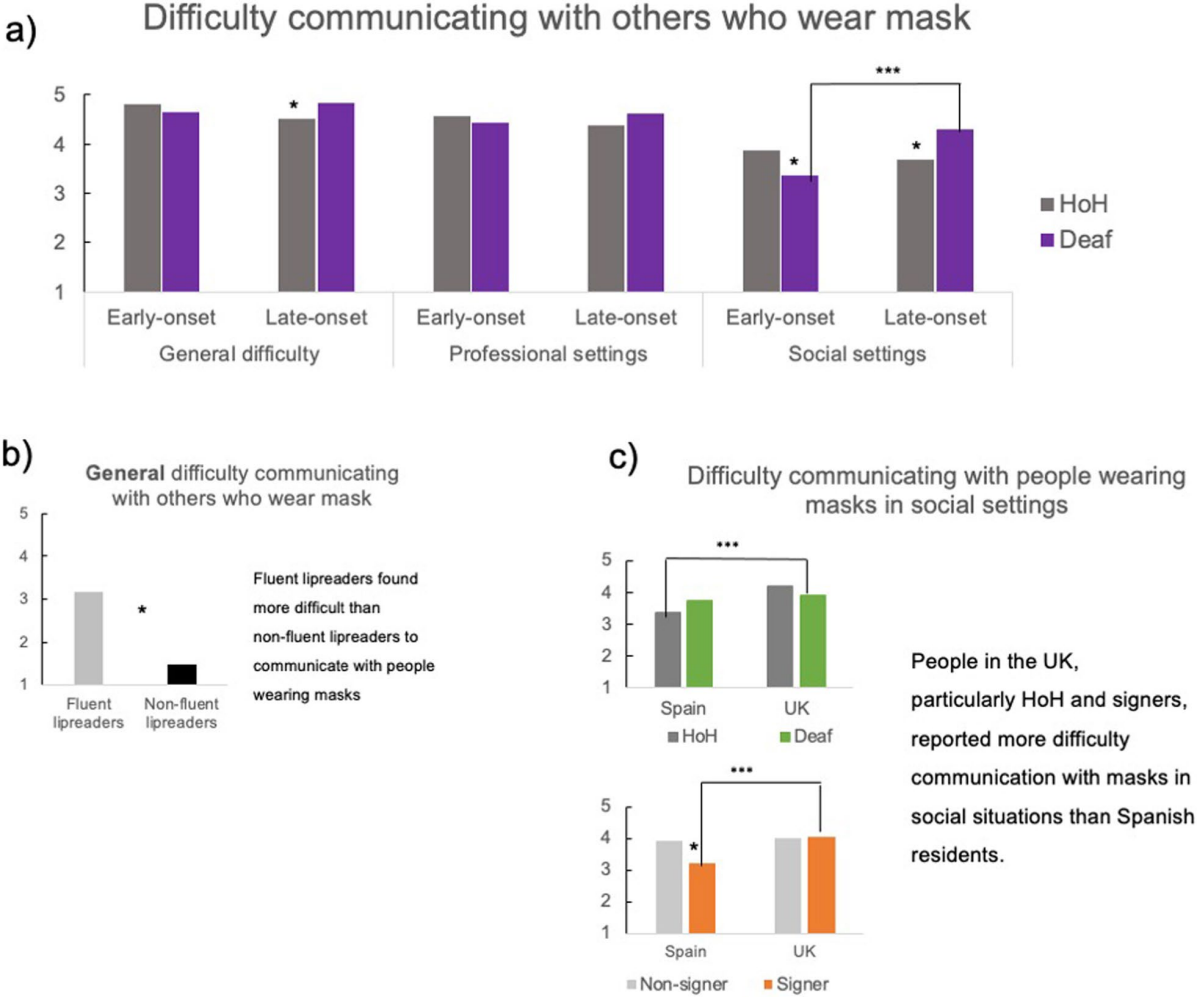
Overview of significant effects for the three measures of experienced difficulty communicating with people who wear masks: Level by onset of deafness interaction in general as well as professional and informal social situations (**a**), main effect of lipreading fluency in the general difficulty measure (**b**) and country of residence by level of deafness and Knowledge of SL interactions for the informal social settings (**c**). Effects that were significant in the simple slopes analysis are coded as follows, *** = *p* < .001, ** = *p* > .01, * = *p* < .05, and +  = *p* < .10

Table 4 and Fig. 3 summarise the significant effects found in this section.

#### General difficulty communicating with people wearing masks

The main effect of lipreading fluency in the main effects model indicated that fluent lipreaders found it more difficult than non-fluent lipreaders to communicate with people wearing masks in general.

The single slope analyses of the level of deafness by onset of deafness interaction in the 2-way interaction model showed that people who became deaf later in life experienced more difficulty if they were deaf than if they were HoH, *b* = 0.224, *t*(367) = 2.175, *p* = 0.030, 95% CI [0.015, 0.300]. This was not the case for those with early-onset deafness (*p* > 0.2).

The single slope analyses of the country of residence by lipreading fluency interaction in the 2-way interaction model revealed a trend for fluent lipreaders in the UK to experience more mean difficulty than those living in Spain, *b* = 0.203, *t*(367) =  − 1.935, *p* = 0.054, 95% CI [− 0.280, 0.002], this was not significant in non-fluent lipreaders (*p* > 0.1).

Overall, people who had become deaf later in life experienced more general difficulty communicating with people who were wearing masks both if they were deaf and if they were HoH. Furthermore, fluent speechreaders, particularly in the UK, struggled to a greater extent.

#### Difficulty communicating with people who wear masks in professional settings

The main effect of country of residence in the main effects-only model *indicated* that UK residents found more difficult than Spanish residents to communicate with people wearing masks in professional settings.

The single slope analyses of the significant interactions on the 2-way and 3-way models showed significant simple effects only for the lipreading fluency by country of residence interaction in the 3-way interaction model (all other *p*s > 0.1). Specifically, for fluent lipreaders UK residents (1) perceived communicating with people using masks in professional settings more difficult than Spanish residents (− 1), *b* = 0.154, *t*(366) = 2.119, *p* = 0.035, 95% CI 0.008, 0.222], this was not significant for non-fluent lipreaders (*p* > 0.05).

Overall, people in the UK, particularly fluent lipreaders, found it more difficult to communicate with people wearing masks in professional settings than people living in Spain but there were no other significant differences.

#### Difficulty communicating with people wearing masks in informal social situations

The significant effects in the main effects only model showed that early-onset deaf people experienced less difficulty than people who became deaf later in life, signers experienced less difficulty than non-signers, and fluent lipreaders experienced more difficulty than non-fluent lipreaders.

The simple slope analyses of the level of deafness by onset of deafness interaction in the 2-way interaction model showed that deaf people who became deaf early in life experienced less difficulty than people who became deaf later in life, *b* = − 0.368, *t*(356) =  − 3.719, *p* < 0.001, 95% CI − 0.702, − 0.216], this was not significant for HoH people (*p* > 0.4). In addition, for participants with early onset deafness, deaf participants experienced less difficulty than HoH participants, *b* = − 0.193, *t*(356) =  − 2.241, *p* = 0.026, 95% CI − 0.467, − 0.030]. The opposite pattern was found for late deafness onset people, HoH people experienced more difficulty than deaf people, *b* = 0.232, *t*(356) = 2.462, *p* = 0.014, 95% CI 0.060, 0.537].

The simple slope analyses of the country of residence by level of deafness interaction in the 2-way interaction model showed that HoH people living in the UK experienced more difficulty compared to those living in Spain, *b* = 0.330, *t*(356) = 3.465, *p* < 0.001, 95% CI 0.179, 0.650], this was not significant for deaf people (*p* > 0.401).

The simple slope analyses of the country of residence by knowledge of SL interaction in the 2-way interaction model showed that residents in Spain who know SL experienced significantly less difficulty than non-signers, *b* = − 0.275, *t*(356) =  − 2.150, *p* = 0.032, 95% CI − 0.676, − 0.030], this was not the case for UK residents, (*p* > 0.8). In addition, signers who lived in the UK experienced relatively more difficulty than signers who lived in Spain, *b* = 0.348, *t*(356) = 3.910, *p* < 0.001, 95% CI 0.217, 0.656], this was not the case for non-signers (*p* > 0.05).

Overall, people who became deaf later in life, especially the HoH people, and fluent lipreaders experienced more difficulty communicating with people wearing masks in informal social situations. Furthermore, people in the UK reported communication with people using masks in informal social situations more difficult than Spanish residents. Indeed, Spanish signers reported less difficulties in communication in informal social situations.

### B. Loss of information and wellbeing

**Fig. 4:**
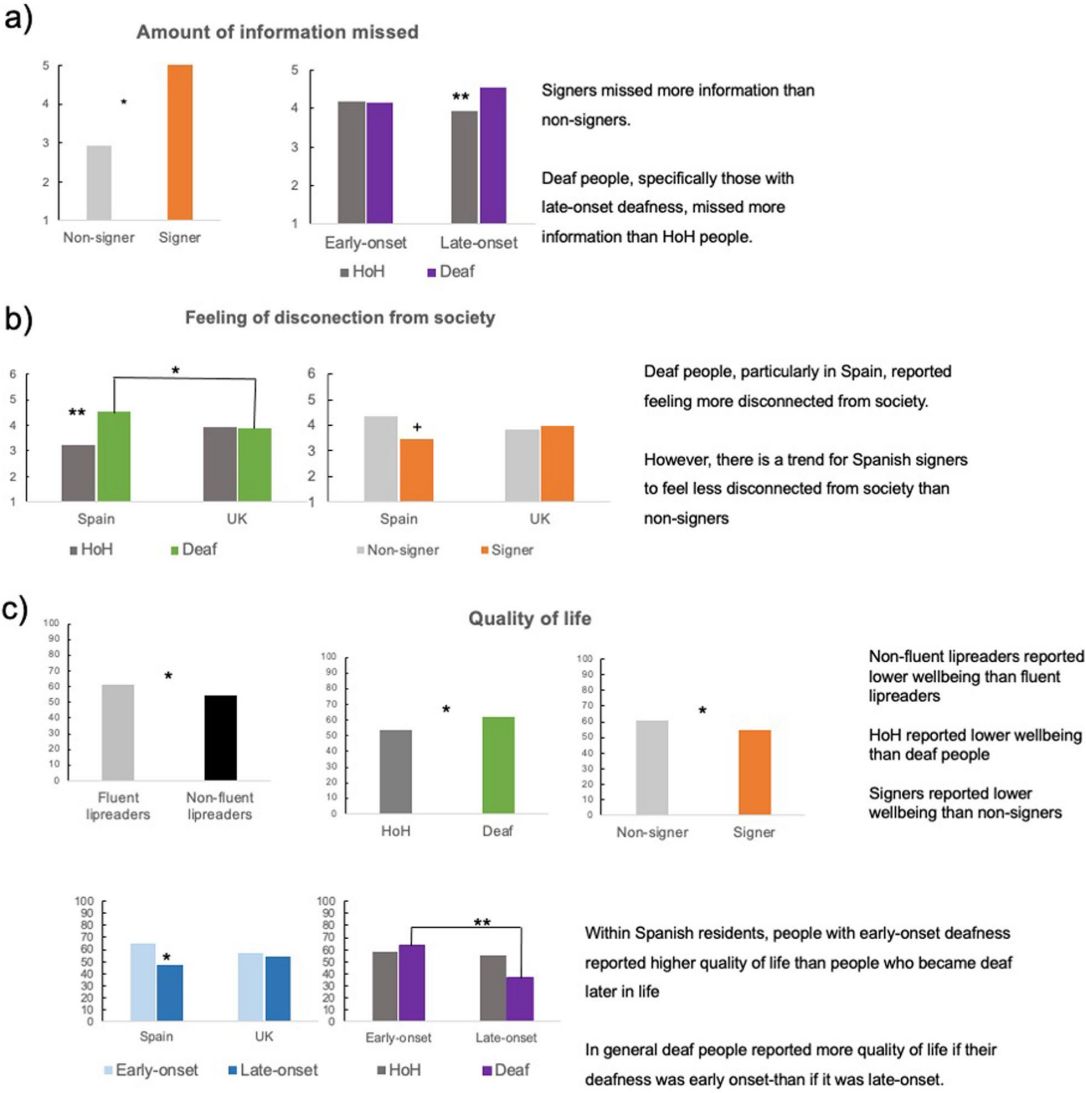
Overview of significant effects for the three measures of perceived loss of information and wellbeing: amount of information missed (**a**), feeling of disconnection from society (**b**) and general wellbeing: quality of life (**c**). Effects that were significant in the simple slopes analysis are coded as follows, *** = *p* < .001, ** = *p* > .01, * = *p* < .05, and +  = *p* < .1

Table 5 and Fig. 4 summarise the significant effects found for Amount of information missed, feeling of disconnection from society, and general wellbeing.

#### Amount of information missed

The significant effects in the main effects only model showed that HoH people (− 1) missed less information that deaf people (1; *p* = 0.026), and signers (1) tend to miss more information than non-signers (− 1; *p* = 0.054).

The simple slope analyses of the level of deafness by onset of deafness interaction in the 2-way interaction model showed that for people with late-onset deafness, deaf people missed more information than HoH people, *b* = − 0.274, *t*(358) = 2.686, *p* = 0.008, 95% CI 0.081, 0.524], this was not the case for early onset deafness (*p* > 0.8).

Overall, signers and deaf, specifically late-onset deaf people, reported to have missed more information.

#### Emotional wellbeing: feeling of being disconnected from society

The main effect of deafness in the 2-way and the 3-way interaction model showed that deaf people (1) felt more disconnected from society than HoH people (− 1; *p* = 0.009).

The simple slope analyses of the country of residence by level of deafness interaction in the 2-way interaction model showed that deaf people living in Spain felt more disconnected from society than UK residents, *b* = − 0.202, *t*(365) =  − 2.288, *p* = 0.023, 95% CI − 0.632, − 0.048], this was not significant for HoH people (*p* > 0.06). In addition, in Spain HoH people felt less disconnected from society than deaf people, *b* = 0.376, *t*(365) = 2.983, *p* = 0.003, 95% CI [0.222, 1.080], this was not significant for UK residents (*p* > 0.8).

The simple slope analyses of the country of residence by knowledge of SL interaction in the 2-way and the 3-way interaction models showed a trend for residents in Spain who know SL to feel less disconnected from society than non-signers, *b* = − 0.264, *t*(365) =  − 1.874, *p* = 0.062, 95% CI − 0.939, 0.022], this was not the case for UK residents (*p* > 0.5).

The simple slope analyses of the 3-way interaction between level of deafness, onset of deafness, and knowledge of SL showed that none of the simple effects reached significance (all *p*s > 0.5).

Overall, deaf people reported feeling more disconnected from society. However, there is a trend for Spanish signers to feel less disconnected from society than non-signers.

#### General wellbeing: quality of life

Significant effects in the main effects-only model revealed that HoH people reported worst wellbeing than deaf people (*p* = 0.018), signers reported lower wellbeing than non-signers (*p* = 0.010), and non-fluent lipreaders reported lower wellbeing than fluent lipreaders (*p* = 0.038).

The simple slope analyses of the country of residence by onset of deafness interaction in the 2-way interaction model showed that for Spanish residents early-onset people (1) reported higher quality of life than people who became later in life (− 1; *b* = 0.384, *t*(367) = 2.583, *p* = 0.010, 95% CI 2.086, 15.405]), this was not the case for UK residents (*p* > 0.4).

The simple slope analyses of the level of deafness by onset of deafness interaction in the 3-way interaction model showed that for deaf people those who became deaf early in life (1) experienced more quality of life than people who became deaf later in life (− 1), *b* = 0.339, *t*(367) = 3.131, *p* = 0.002, 95% CI 2.897, 12.679], this was not significant for HoH people (*p* > 0.3).

The simple slope analyses of the country of residence by level of deafness interaction in the 3-way interaction model showed no significant simple effects (all *p*s = 0.07).

Overall, HoH people, deaf people with late-onset deafness, non-fluent lipreaders, and signers reported the worst general wellbeing.

### C. Ways to improve communication

**Fig. 5:**
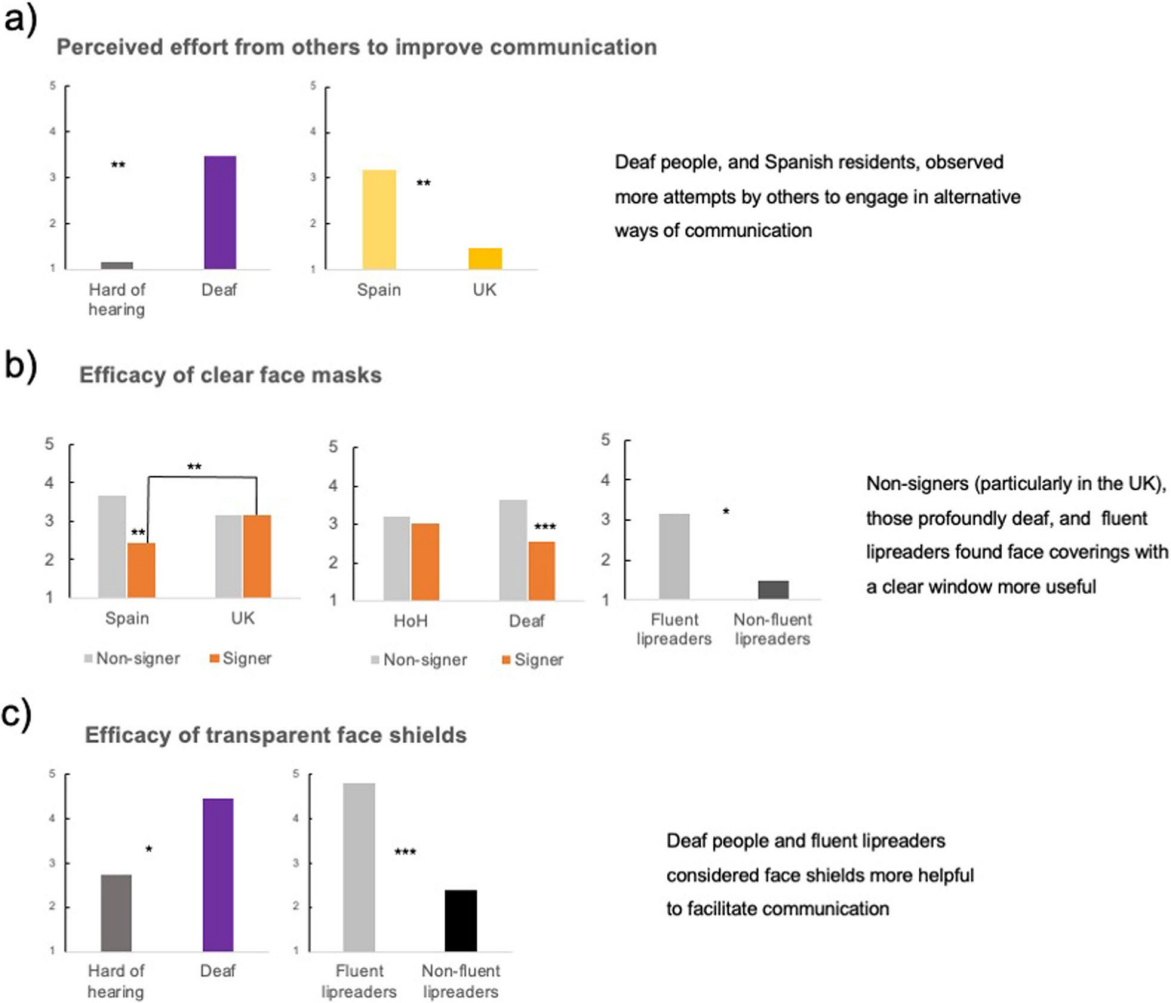
Overview of significant effects for the three measures on respondent’s opinion on ways to improve communication: other’s efforts to engage in alternative communication such as gesturing or writing (**a**), efficacy of face masks with a clear window (**b**), and efficacy of transparent face shields (**c**). Effects that were significant in the simple slopes analysis are coded as follows, *** = *p* < .001, ** = *p* > .01, * = *p* < .05, and +  = *p* < .1

Table 6 and Fig. 5 summarise the significant effects found for the outcome variables included in this section.

#### Perceived effort from others to improve communication

Significant effects in the main effects-only model revealed that deaf people reported that others have made more efforts to communicate with them while wearing a mask than HoH people (*p* = 0.016). Residents in the UK had experienced that other people had done fewer extra efforts to communicate with them (*p* = 0.009).

The analysis of the simple slopes of the country of residence by onset of deafness interaction in the 3-way interaction model showed a trend for UK residents to report that other had done less efforts to communicate with them than for Spanish residents, *b* = − 0.171, *t*(356) =  − 1.949, *p* = 0.052, 95% CI − 0.403, 0.002] for people who became deaf early in life but not for people with late-onset deafness (*p* > 0.4).

Overall, deaf people, and Spanish residents reported having observed more attempts by others to engage in alternative ways of communication such as writing or gesturing.

#### Perceived efficacy of transparent or clear window masks to facilitate communication

Significant effects in the main effects-only model revealed that signers (1) perceived clear window masks as less useful than non-signers (− 1; *p* = 0.003), and fluent lipreaders (1) thought that clear windows masks were more useful than non-fluent lipreaders (− 1; *p* = 0.02).

The analysis of the simple slopes of the country of residence by knowledge of SL interaction in the 2-way interaction model showed that for Spanish residents, non-signers perceived transparent masks as more useful than signers, *b* = − 0.439, *t*(368) =  − 3.194, *p* = 0.002, 95% CI [− 0.997, − 0.237], this was not the case for UK residents (*p* > 0.9). Additionally, signers who lived in the UK reported that clear window masks were more useful than signers in Spain, *b* = 0.260, *t*(368) = 2.711, *p* = 0.007, 95% CI [0.098, 0.614].There were no differences for non-signers (*p* > 0.05).

The analysis of the simple slopes of the level of deafness by knowledge of SL interaction in the 2-way interaction model showed that deaf people who do not know SL perceived transparent masks as more useful than signers, *b* = − 0.388, *t*(368) =  − 3.887, *p* < 0.001, 95% CI [− 0.822, − 0.270], this was not the case for HoH people (*p*s > 0.091).

There were no other significant simple effects for the significant interaction in the 3-way model (all *p*s > 0.174).

Overall, non-signers and fluent lipreaders found face coverings with a clear window more useful.

#### Perceived efficacy of transparent face shields to facilitate communication

Significant effects in the main effects-only model revealed that HoH people (− 1) considered face shields as less helpful to facilitate communication than deaf people (1; *p* = 0.049). Fluent lipreaders (1) valued the transparent face shields as more positive than less fluent lipreaders (− 1; *p* = 0.001).

Overall, deaf people and fluent lipreaders found transparent face shields more useful.

## Discussion

The present study was designed to explore the communication difficulties experienced by deaf and HoH people due to the use of face coverings in the current COVID-19 pandemic. Furthermore, we studied which factors predicted the amount of information they had missed since the start of the pandemic as well as their emotional and general wellbeing. Finally, we explored deaf and HoH people’s views on three different ways in which communication could be improved: alternative ways of communicating, use of face masks with a clear window, and use of transparent face shields. We included level and onset of deafness, knowledge of SL, self-assessed speechreading fluency, and country of residence as predictors in all the analyses.

By focusing on accessibility, the present study addresses important limitations present in prior research. We increased accessibility by releasing the same survey questions not only in written English and Spanish but also in three different sign languages (BSL, LSE, and LSC). This was done to achieve a better representation of the deaf population, including deaf/HoH people whose first language is one of the three studied sign languages. Providing all participants with the option of seeing the survey items in their natural language has the added benefit of including signers that have little access to written language. This is important given that a majority of deaf people find reading a challenging task, with most leaving school having achieved a reading age of about 8^th^ grade at the most (e.g. Sánchez & García-Rodicio, 2006; Traxler, 2000) or even lower (Domínguez & Alegria, 2010). These signers with lower reading skills are likely to be substantially underrepresented in surveys that are delivered only in writing. Indeed, approximately one third of our respondents accessed the SL version despite personal costs to them such as taking longer to complete, requiring more bandwidth, and possibly needing a larger device to watch the videos comfortably. This high number of deaf people completing the signed version highlights the importance of dedicating more resources (i.e. funds and time necessary to create adequate SL clips) so sign language is used as a main language in research involving deaf people.

Our results show that the deaf people who answered this survey, as well as their relatives and friends, engaged in health protection behaviours such as mask wearing despite communication challenges. This is likely to have contributed to the small percentage of participants (5.3% across both countries) who had had confirmed or suspected COVID-19 at the time of testing. We found that communication with people who wear masks was difficult for all participants in all situations. Overall respondents felt that they had missed a lot of information in their day-to-day interactions with people. They have also felt significantly more disconnected from society than before. Nonetheless, in general, responses show that deaf and HoH people appreciate that others have made some efforts to communicate in some other way such as gesturing or writing. They also valued positively the use of transparent face coverings. In the rest of this section, we summarise and discuss the key findings separately in each of the three areas of interest: communication difficulties, loss of information and wellbeing, and ways to improve communication.

### Difficulties communicating with people who wear face masks

When examining the general communication difficulties, our findings revealed a key role of speechreading fluency predicting level of difficulty, particularly for UK residents. Overall, fluent speechreaders struggled more than non-fluent speechreaders. This result is not surprising, as it reflects that deaf and HoH people heavily rely on their interlocutor’s lip movements during face-to-face interactions (Atcherson et al., 2017; Moberly et al., 2020; Naylor et al., 2020; Saunders et al., 2021; Trecca et al., 2020), with those who consider themselves fluent speechreaders more likely to rely more on lip patterns than those who are not very fluent.

The analysis examining difficulty of communication when others use mask in professional settings showed a clear effect of country of residence, with UK residents struggling more than Spanish residents. As a reminder, Spanish respondents had spent significantly more time interacting with people wearing masks (independent samples *t*-test: *t*(390) = 5.94, *p* < 0.001). Interestingly, for informal social communication the decrease in difficulty experienced by Spanish residents was specific to HoH (vs. deaf) people and to signers (vs. non-signers).

There are several possible explanations for these effects. One possibility is that people in Spain (deaf and hearing) were making a greater effort to communicate widely because mask-wearing was more ubiquitous, and hence it was hindering communication at some level for everyone. Likewise, the stricter restrictions around mask wearing in Spain (e.g. fewer exceptions were allowed and authorities ensuring that someone not wearing a mask carried an official exemption certificate) could contribute to a more general awareness that others might need additional support in the communication process. This interpretation is supported by the fact that the interlocutors of Spanish residents made more efforts in communicating using gesturing, writing, or some other creative alternatives (see the “ways to improve communication” section in results and below), which could have contributed to decrease the perception that communication was difficult.

A second possibility is that in Spain people generally use more alternative ways of visual communication such as gesture, not just as an increased level of awareness after COVID-19 but as a cultural trait (see Van Deusen Phillips, 2008). Southern Europeans (e.g. Italians, Spaniards, Greeks) are often considered to be immersed in gesture rich cultures (for discussion of cross-cultural differences in gestures see Kendon, 2004; Kita, 2009). For example, Italian children 2 years old and younger used more gestures referring to objects, people or locations (i.e. representational gestures) than American children. The Italian children also used these representational gestures more frequently than the American children (Iverson et al., 2008). American children relied more on their larger speech vocabulary, pointing, and more conventional gestures (e.g. Hi, Yes, All-gone). These differences observed between Italian and American children, could be parallel between Spanish and UK people. This interpretation is consistent with the trend in the general difficulty measure for speechreading fluency to be a stronger predictor of difficulties for UK than for Spanish residents. Within this assumption, a richer and more varied representational gesture inventory could be more helpful when speechreading is not possible. Indeed, gestures and facial expressions have been shown to enhance speech understanding at different levels of noise-vocoding (Drijvers & Özyürek, 2017).

The fact that Spanish signers experienced less difficulty communicating with people who wear masks in informal social settings further supports the idea that visual communication that does not rely primarily on speechreading has a beneficial effect such that facilitates communication.

A third possibility is that other cultural differences such as collectivism contribute to these dissimilarities between UK and Spanish residents’ perceived difficulty. People in collectivistic cultures tend to be part of strong cohesive groups, having closer and more supportive networks (Triandis et al., 1988). For example, Goodwin and Hernandez Plaza (2000) found that Spanish people were more collectivistic than people in the UK. Furthermore, they found that the collectivism predicted Spanish residents perceived that they had received global support both in general and after an event. It is possible that, because Spain is a more collectivistic society than the UK, Spanish people expect and report receiving more social support to overcome communication difficulties. It is also possible that people disadvantaged by communication barriers in the UK simply expect more social support than what they receive. Their higher scores in perceived difficulty could reflect their violated expectations, as violated expectations have been associated with greater difficulty experienced across different contexts (e.g. Belsky, 1985; Burgoon, 1993; Gao, 2020; Violanti, 2020). Previous research has shown that in countries with a lower Power Distance Index (PDI; for comparison see Insights, 2021) such as the UK when compared to Spain, people align more with the belief that inequalities should be minimised and therefore expect more support to minimise them. More research is needed to further understand whether these differences are related to having developed more general awareness of the need to find alternative ways to communicate, or rather, to more general cultural factors such as more extensive use of gestures, or different expectations of how much support the society as a whole should provide.

Regardless of the ultimate cause of the perceived difficulty, our findings indicate that to decrease communication difficulties when wearing masks, we can encourage using sign language when possible and investing effort in alternative visual communication when signing is not possible (for similar recommendation see e.g. Mheidly et al., 2020; Saunders et al., 2021; Sanjeev et al., 2021). Sanjeev et al. (2021) highlighted the advantages (e.g. effective communication) of using sign language in emergency departments, intensive care units, and operating rooms in hospitals, particularly before performing high-risk procedures.

Another compelling finding is that deaf people with late-onset deafness were the group experiencing more difficulties across the board, as indicated by the interactions between onset and degree of deafness. There were two key results that supported this statement. First, people with late-onset deafness experienced more difficulty in general if they were deaf than if they were HoH. This could reflect the fact that late-onset HoH people are still able to use some auditory information in their interactions which could help effective communication to some degree and make the process less difficult. Despite the reduction in quality of the speech signal due to the masks (Atcherson et al., 2017; Trecca et al., 2020), they could still use this degraded auditory information and hence reduce their perception of difficulty. Late-onset deaf people would depend exclusively on speechreading, therefore struggling more when masks are used. Second, deaf people who became deaf later in life experienced more difficulty communicating than deaf people with early-onset deafness, particularly in informal social situations. This increased difficulty for late-onset deaf people indicates that they depend on speechreading more than early-onset deaf people, who are likely to have developed communication strategies involving a wider range of visual information. For example, late-onset deaf people are less likely to learn sign language and consequently less likely to develop a social network around signing (*for discussion about deafhood, how the creation of deaf identity leads to the use of SL and the composition of signing communities see e.g. *Hauser et al., 2010*; *Padden et al., 2009). Indeed, while 87.5% of our early-onset participants were signers, only 36% of the late-onset respondents knew a SL. Other factors different from signing status could explain to a greater extent why late-onset deaf people experience more difficulties. Individuals who became deaf later in life could be more affected by the feelings of shame and the stigma associated with deafness perceived as a disability (Jones, 2002). On the contrary, deaf/HoH people who belong to a deaf community (e.g. early-onset deaf people immersed in deaf culture), are likely to consider deafness as an integral part of their identity and be less influenced by stigma (Bauman & Murray, 2014; Fleischer et al., 2016). More research is needed to investigate whether a stronger deaf identity and/or less stigma results in early-onset deaf people identifying more as deaf when interacting with people, and perhaps changing the communication dynamics. In summary, we propose that late-onset deaf people are less likely to benefit from a social network of people who sign and form a perception of deafness as part of naturally occurring diversity rather than as a disability that causes shame. Both, the lack of social network of signers and the negative perception of deafness, could contribute to more difficult communication.

### Loss of information and wellbeing

The findings discussed so far identified fluent speechreaders and late-onset deaf people as the groups who found communication with masks more difficult. Additionally, we found that some signers (Spanish) reported communication with people who wear masks being less difficult. However, when we explored the possible detrimental consequences of communication using face masks on important information missed and wellbeing, the data did not completely align with this pattern. In agreement with the difficulty measures, late-onset deaf people had lower general wellbeing scores. However, all deaf people, including both late- and early-onset, reported having missed more information due to the use of face masks in their interactions with people and feeling more disconnected from society. These results indicate that it is the degree of deafness, rather than the onset, that bears a heavier weight on loss of information while communicating with face coverings.

Signers reported having missed a substantial amount of information in face-to-face interactions where the interlocutor wore a mask. Signers also reported lower general wellbeing. However, the group of Spanish signers felt less disconnected from society. This finding might be related to their informal social interactions using SL and to an increase in accessible signed material during the pandemic. Indeed, deaf organisations and individuals increased their production of signed resources. For example, the Spanish national confederation of deaf people (Confederacion Estatal de personas sordas: CNSE), published over one hundred COVID-19 related videos in LSE between January 2020 and July 2021. Finally, many deaf individuals offered signed resources aimed to keep signers both informed and entertained while restrictions on face-to-face meetings were in place. Many of these individual webpages were extremely popular. For example, the webpage https://encasa.excepcionales.es/ received over 10,000 visits between March and December 2020. The sudden increase in signed resources from the deaf communities could have resulted in a reduced feeling of disconnection from society. However, this does not explain differences between Spanish and UK signers, because similar increases in the signed resources available were observed in the UK. For example, deaf schools (e.g. Frank Barnes or Blanche Nevile) developed numerous resources for children. The Royal Association for Deaf people (RAD) and organisations such as Sign Health or Deaf Station regularly provided updates about the news, COVID-19 related information, and so on. More research is needed to fully understand the differences between the two countries. It is possible that the Spanish society, being more collectivistic (Goodwin & Hernandez Plaza, 2000) than UK’s society, behaved more pro-socially (for further support of this argument see e.g. Feygina & Henry, 2015), reducing the feeling of disconnection from society.

### Ways to improve communication

Regarding the ways in which communication can be improved while staying safe through the use of masks, we found the following key results:

First, deaf people reported more alternative efforts of their interlocutors to communicate, for example writing or gesturing, than HoH people. Second, Spanish residents also reported that others had made more extra efforts to communicate than UK residents. As discussed at length above there are two possible explanations for differences between the two countries. One is that cultural differences in the type of gesture and non-verbal communication result on Spanish people being more used to frequent use of meaningful gesturing. Another possibility is cultural differences in collectivism/individualism are related to increased prosocial behaviour in the more collectivist Spanish society. Further research is needed to identify the specific contribution of these factors.

Third, fluent speechreaders found useful both masks with a transparent (clear) window and completely transparent face-shields. This result is congruent with the wealth of research showing that people (both deaf and hearing) pay close attention to the mouth when trying to understand speech, particularly when auditory perception becomes difficult (e.g. perception in noise, vocoded speech, or when listening to an unfamiliar language, see e.g. Banks et al., 2021; Blackburn et al., 2019; Lusk & Mitchel, 2016; Worster et al., 2018).

Fourth, deaf people, including signers and non-signers, found transparent face shields useful. It is possible that HoH people do not find transparent face shields as useful because of the sound attenuation, which is greater for clear masks and transparent face shields than for surgical masks.^2^ The harder materials of the face shields could hinder their residual hearing more than the clear face masks. However, further research specifically aimed to test this hypothesis is needed before drawing conclusions. Interestingly, it was non-signers who found clear window face masks more useful than signers, suggesting that for non-signers just seeing the lips is useful while signers might appreciate more seeing the whole facial expression. However, it is well-know that there are issues regarding cost, manufacturing, and safety of completely transparent face coverings. We propose that when they are used to facilitate communication with signers, other factors such as ventilation and increased social distancing are put in place to ensure safety.

The limitations of this study include the longer length of the survey due to the exploratory nature of this study. A shorter survey could have attracted higher participation. The process of translation to multiple SL, video editing, and familiarisation with releasing surveys using video content delayed the release of the survey or perhaps early experiences were not completely captured in this work. It is worth to note that the increased accessibility due to the release in several sign languages, as well as the recruitment through the deaf communities’ channels, meant that the proportion of signers who participated in this study might not be representative of the proportion of signers in the general population. This is not a limitation per se, as we aimed to over-recruit from this population. Finally, the fact that the survey was online made it accessible internationally and easy to participate at any time convenient. However, due to this online nature, some older people as well as deaf people with lower technological literacy are likely to be unrepresented. Further research would need to be conducted on those groups, perhaps with an interviewer that can facilitate the interaction with the survey. Similarly, it is possible that some deaf COVID-19 patients did not take part in this study due to their poor health. It is also possible that deaf people who contracted COVID-19 and did not participate in the present study were not strong mask users. In light of the present results, further research is needed to completely disentangle the effects of mask use frequency from other factors, including the amount of time they spent communicating in SL in comparison with spoken language, SL proficiency, as well as the other possible factors discussed above.

## Conclusions

The complex pattern of results found here highlights the importance of acknowledging the diversity of deaf/HoH people when studying the impact of community-wide use of face masks. Rather than finding a solution that fits all deaf/HoH people, solutions and interventions should be tailored to different experiences due to onset and level of deafness, signing status, and the specific person’s speechreading fluency. We also found that the effects of mask-wearing frequency might be modulated by cultural factors such as generalised use of gesturing, pro-social behaviour (e.g. generalised willingness to use gestures or other non-verbal communication), or people’s perception of how much power they hold in society. Finally, our findings revealed that clear masks are more useful than standard masks for everyone. However, seeing the lip patterns is not all that there is. On the one hand, seeing the whole face and expression is important for some deaf/HoH people. On the other hand, communication partners using sign language, gestures, and other ways of non-verbal communication can help many deaf/HoH people.

## Supplementary Information

**Additional file 1: **Full statistics for the regression analyses, including simple effects analyses of the significant interactions.

## Data Availability

The datasets generated and analysed during the current study, as well as the regression analyses code, are available in the OSF repository https://osf.io/5rtpj/?view_only=374488bf57be40698b4c14e80137ff32.

## Abbreviations

HoH: Hard of hearing
SL: Sign language
BSL: British Sign Language
LSE: Lengua de Signos Española
LSC: Llengua de Signes Catalana

## Footnotes

1 Speechreading refers to comprehension of speech through vision. Lipreading is a main component of speechreading and a highly familiar term. The present study used the term lipreading in the survey questions to maximise understanding by a heterogeneous pool of respondents. Therefore, we used the label lipreading fluency for the related dependent measure for precision. However, throughout the introduction and discussion we used the term speechreading to acknowledge the fact that deaf people usually take cues from the whole face rather than just the lips.

2 This assumes that completely transparent face coverings are worn on their own.

## References

[CR1] Aiken, L. S., West, S. G., & Reno, R. R. (1991). *Multiple regression: Testing and interpreting interactions*. sage.

[CR2] Atcherson SR, Mendel LL, Baltimore WJ, Patro C, Lee S, Pousson M, Spann MJ (2017). The effect of conventional and transparent surgical masks on speech understanding in individuals with and without hearing loss. Journal of the American Academy of Audiology.

[CR3] Ballard, J. (2022, Februeary 23). *From pandemic to endemic: The future of masking*. Nwes. Faculty & Staff, Researhc & Scholarship. University of Denver. Retrieved from, https://www.du.edu/news/pandemic-endemic-future-masking

[CR4] Banks B, Gowen E, Munro K, Adank P (2021). Eye gaze and perceptual adaptation to audiovisual degraded speech. Journal of Speech, Language, and Hearing Research..

[CR5] Barker AB, Leighton P, Ferguson MA (2017). Coping together with hearing loss: A qualitative meta-synthesis of the psychosocial experiences of people with hearing loss and their communication partners. International Journal of Audiology.

[CR6] Bauman H-DL, Murray JJ (2014). Deaf gain: Raising the stakes for human diversity.

[CR7] BBC. (2021, March 21). *Covid: Masks and social distancing 'could last years'*. Retrieved from, https://www.bbc.co.uk/news/uk-56475807

[CR8] Belsky J (1985). Exploring individual differences in marital change across the transition to parenthood: The role of violated expectations. Journal of Marriage and the Family.

[CR9] Blackburn CL, Kitterick PT, Jones G, Sumner CJ, Stacey PC (2019). Visual speech benefit in clear and degraded speech depends on the auditory intelligibility of the talker and the number of background talkers. Trends in Hearing.

[CR10] Burgoon JK (1993). Interpersonal expectations, expectancy violations, and emotional communication. Journal of Language and Social Psychology.

[CR11] Chodosh J, Weinstein BE, Blustein J (2020). Face masks can be devastating for people with hearing loss. British Medical Journal Publishing Group.

[CR12] Chu DK, Akl EA, Duda S, Solo K, Yaacoub S, Schünemann HJ, COVID-19 Systematic Urgent Review Group Effort (SURGE) study authors (2020). Physical distancing, face masks, and eye protection to prevent person-to-person transmission of SARS-CoV-2 and COVID-19: A systematic review and meta-analysis. The Lancet.

[CR13] Van Deusen Phillips, S. B. (2008). *When looking is listening: Gesture, multi-modal language, and the socialization of deaf children in castellano-speaking families*. (Ph.D.). The University of Chicago, Ann Arbor.

[CR14] Domínguez AB, Alegria J (2010). Reading mechanisms in orally educated deaf adults. Journal of Deaf Studies and Deaf Education.

[CR15] Drijvers L, Özyürek A (2017). Visual context enhanced: The joint contribution of iconic gestures and visible speech to degraded speech comprehension. Journal of Speech, Language, and Hearing Research.

[CR16] Emilio Ferreiro [@emilioferreiro]. (2020). *La campaña pidiendo mascarillas transparentes para personas sordas, ha perdido totalmente el norte. La mascarilla transparente es necesaria pero NO es suficiente y está enviando un mensaje social erróneo y peligroso*. Twitter. Retrieved from, https://twitter.com/emilioferreiro/status/1306613700883775488

[CR17] Epifanio MS, Andrei F, Mancini G, Agostini F, Piombo MA, Spicuzza V, Riolo M, Lavanco G, Trombini E, La Grutta S (2021). The impact of COVID-19 pandemic and lockdown measures on quality of life among Italian general population. Journal of Clinical Medicine.

[CR18] Feygina I, Henry PJ, Schroeder DA, Graziano WG (2015). Culture and prosocial behavior. The Oxford handbook of prosocial behavior.

[CR19] Fleischer FS, Garrow WG, Bauman H-DL, Murray JJ (2016). Review of *Deaf gain: Raising the stakes for human diversity*. Sign Language Studies.

[CR20] Gao, Y. (2020). *All is not lost: How negative life events help emerging adults achieve life satisfaction?* (Doctoral dissertation, Villanova University).

[CR21] Garstecki DC, Erler SF (1999). Older adult performance on the communication profile for the hearing impaired: Gender difference. Journal of Speech, Language, and Hearing Research.

[CR22] Goodwin R, Hernandez Plaza S (2000). Perceived and received social support in two cultures: Collectivism and support among British and Spanish students. Journal of Social and Personal Relationships.

[CR23] Gravel JS, O'Gara J (2003). Communication options for children with hearing loss. Mental Retardation and Developmental Disabilities Research Reviews.

[CR24] Hauser PC, O’Hearn A, McKee M, Steider A, Thew D (2010). Deaf epistemology: Deafhood and deafness. American Annals of the Deaf.

[CR25] Henn P, O’Tuathaigh C, Keegan D, Smith S (2021). Hearing impairment and the amelioration of avoidable medical error: A cross-sectional survey. Journal of Patient Safety.

[CR26] Homans NC, Vroegop JL (2022). The impact of face masks on the communication of adults with hearing loss during COVID-19 in a clinical setting. International Journal of Audiology.

[CR27] Howard J, Huang A, Li Z, Tufekci Z, Zdimal V, van der Westhuizen HM, von Delft A, Price A, Fridman L, Tang LH, Tang V, Watson GL, Bax CE, Shaikh R, Questier F, Hernandez D, Chu LF, Ramirez CM, Rimoin AW (2021). An evidence review of face masks against COVID-19. Proceedings of the National Academy of Sciences.

[CR28] Humphries T, Kushalnagar P, Mathur G, Napoli DJ, Padden C, Rathmann C, Smith SR (2012). Language acquisition for deaf children: Reducing the harms of zero tolerance to the use of alternative approaches. Harm Reduction Journal.

[CR29] Ideas for ears. (2020). *Survey results—Impact of masks, 2m distancing & more.* Retrieved from, https://www.ideasforears.org.uk/blog/survey-results-impact-or-masks-and-more/

[CR30] INE. (2021). Retrieved from, https://www.ine.es/dyngs/INEbase/es/operacion.htm?c=Estadistica_C&cid=1254736176782&menu=resultados&idp=1254735573175

[CR31] Hoftede Insights. (2021).*Country comparison*. Retrieved from, https://www.hofstede-insights.com/country-comparison/spain,the-uk/

[CR32] Iverson JM, Capirci O, Volterra V, Goldin-Meadow S (2008). Learning to talk in a gesture-rich world: Early communication in Italian vs. American Children. First Language.

[CR33] jewishnews.timesofisrael.com. (2020, April 23). *Masks-for-all rule would be ‘disaster’ for deaf people*. Retrieved from, https://jewishnews.timesofisrael.com/masks-for-all-rule-would-be-disaster-for-deaf-people/

[CR34] Jones M (2002). Deafness as culture: A psychosocial perspective. Disability Studies Quarterly.

[CR35] Kendon A (2004). Gesture: Visible action as utterance.

[CR36] Kita S (2009). Cross-cultural variation of speech-accompanying gesture: A review. Language and Cognitive Processes.

[CR37] Kushalnagar P, Mathur G, Moreland CJ, Napoli DJ, Osterling W, Padden C, Rathmann C (2010). Infants and children with hearing loss need early language access. The Journal of Clinical Ethics.

[CR38] Kyle FE, Campbell R, Mohammed T, Coleman M, MacSweeney M (2013). Speechreading development in deaf and hearing children: Introducing the test of child speechreading. Journal of Speech, Language and Hearing Research.

[CR39] Lavanguardia.com (2021, Januray 5). **¿**Cómo afecta el coronavirus a las personas sordas o con problemas de audición?. Retrieved from, https://www.lavanguardia.com/vida/junior-report/20200925/483633951276/como-afecta-coronavirus-personas-sordas-problemas-audicion.html

[CR40] Lusk LG, Mitchel AD (2016). Differential gaze patterns on eyes and mouth during audiovisual speech segmentation. Frontiers in Psychology.

[CR41] Margaret du Feu M (2014). Mental health and deafness.

[CR42] Mayberry RI, Kluender R (2018). Rethinking the critical period for language: New insights into an old question from American Sign Language. Bilingualism: Language and Cognition.

[CR43] Mheidly N, Fares MY, Zalzale H, Fares J (2020). Effect of face masks on interpersonal communication during the COVID-19 pandemic [perspective]. Frontiers in Public Health.

[CR44] Middleton A, Turner GH, Bitner-Glindzicz M, Lewis P, Richards M, Clarke A, Stephens D (2010). Preferences for communication in clinic from deaf people: A cross-sectional study. Journal of Evaluation in Clinical Practice.

[CR45] Moberly AC, Vasil KJ, Ray C (2020). Visual reliance during speech recognition in cochlear implant users and candidates. Journal of the American Academy of Audiology.

[CR46] Mohammed T, Campbell R, Macsweeney M, Barry F, Coleman M (2006). Speechreading and its association with reading among deaf, hearing and dyslexic individuals. Clinical Linguistics & Phonetics.

[CR47] Naylor G, Burke LA, Holman JA (2020). Covid-19 lockdown affects hearing disability and handicap in diverse ways: A rapid online survey study. Ear and Hearing.

[CR48] NDCS. (2020, September 23). *Keep it clear—Survey results on your experience of face masks*. Retrieved from, https://www.ndcs.org.uk/blog/survey-results-face-masks-deaf-children-young-people/

[CR49] NDCS. (2021, February 25). *Face coverings in education—National Deaf Children’s Society position paper*. Retrieved from, https://www.ndcs.org.uk/media/6209/face-covering-in-education-position-paper.pdf

[CR50] Northern JL, Downs MP (2002). Hearing in children.

[CR51] Olusanya BO, Davis AC, Hoffman HJ (2019). Hearing loss grades and the International classification of functioning, disability and health. Bulletin of the World Health Organization.

[CR52] ONS (2018). *Population estimates for the UK, England and Wales, Scotland and Northern Ireland: Mid-2018.* Retrieved from, https://www.ons.gov.uk/peoplepopulationandcommunity/populationandmigration/populationestimates/bulletins/annualmidyearpopulationestimates/mid2018

[CR53] Padden C, Humphries T, Padden C (2009). Inside deaf culture.

[CR54] Ping W, Zheng J, Niu X, Guo C, Zhang J, Yang H, Shi Y (2020). Evaluation of health-related quality of life using EQ-5D in China during the COVID-19 pandemic. PLoS ONE.

[CR55] RNID. (2021a). *Facts and figures*. Retrieved from, https://rnid.org.uk/about-us/research-and-policy/facts-and-figures/

[CR56] RNID. (2021b, November 29th). *Face coverings in England: Deaf people must not be excluded as restrictions change*. Retrieved from, https://rnid.org.uk/2021b/11/new-rules-on-face-coverings-in-england-including-deaf-people-and-people-with-hearing-loss/

[CR57] Saiz MLE, Barberà G, González-Montesino RH, Segimón SF (2022). L’impacte de la COVID-19 en la comunitat sorda: El cas de la llengua de signes espanyola (LSE) i la llengua de signes catalana (LSC). Revista De Llengua i Dret.

[CR58] Sánchez E, García-Rodicio H (2006). Re-lectura del estudio PISA: Qué y cómo se evalúa e interpreta el rendimiento de los alumnos en la lectura. Revista De Educación.

[CR59] Sanjeev OP, Mishra US, Singh A (2021). Sign language can reduce communication interference in Emergency Department. The American Journal of Emergency Medicine.

[CR60] Saunders GH, Jackson IR, Visram AS (2021). Impacts of face coverings on communication: An indirect impact of COVID-19. International Journal of Audiology.

[CR61] Smiljanić R, Bradlow AR (2009). Speaking and hearing clearly: Talker and listener factors in speaking style changes. Language and Linguistics Compass.

[CR62] Specsavers. (2021). *Understanding how face masks impact people with hearing loss*. Retrieved from, https://www.specsavers.co.uk/covid19-care/face-masks-hearing-loss-impacts-and-advice

[CR63] Sutton-Spence R, Woll B (1999). The linguistics of British Sign Language: An introduction.

[CR64] Tavanai E, Rouhbakhsh N, Roghani Z (2021). A review of the challenges facing people with hearing loss during the COVID-19 outbreak: Toward the understanding the helpful solutions. Auditory and Vestibular Research.

[CR65] Tomblin JB, Harrison M, Ambrose SE, Walker EA, Oleson JJ, Moeller MP (2015). Language outcomes in young children with mild to severe hearing loss. Ear and Hearing.

[CR66] Traxler CB (2000). The Stanford Achievement Test: National norming and performance standards for deaf and hard-of-hearing students. Journal of Deaf Studies and Deaf Education.

[CR67] Trecca EM, Gelardi M, Cassano M (2020). COVID-19 and hearing difficulties. American Journal of Otolaryngology.

[CR68] Triandis HC, Bontempo R, Villareal MJ, Asai M, Lucca N (1988). Individualism and collectivism: Cross-cultural perspectives on self-ingroup relationships. Journal of Personality and Social Psychology.

[CR69] Vilagut G, Valderas JM, Ferrer M, Garin O, López-García E, Alonso J (2008). Interpretation of SF-36 and SF-12 questionnaires in Spain: Physical and mental components. Medicina Clinica.

[CR70] Violanti MT (2020). Avoiding expectancy violations: Starting with course content on day 1. Communication Teacher.

[CR71] Ware JE, Kosinski M, Keller SD (1996). A 12-item short-form health survey: Construction of scales and preliminary tests of reliability and validity. Medical Care.

[CR72] WHO. (2021, April 1). *Deafness and hearing loss.* Retrieved from, https://www.who.int/news-room/fact-sheets/detail/deafness-and-hearing-loss

[CR73] Wisconsin Public Radio. (2022, March 15). *Before 2020, they had never worn masks*. *Now, they plan to wear them long into the future*. Retrieved from, https://www.wpr.org/2020-they-had-never-worn-masks-now-they-plan-wear-them-long-future

[CR74] Woodward MF, Barber CG (1960). Phoneme perception in lipreading. Journal of Speech and Hearing Research.

[CR75] Worster E, Pimperton H, Ralph-Lewis A, Monroy L, Hulme C, MacSweeney M (2018). Eye movements during visual speech perception in deaf and hearing children. Language Learning.

